# *MC4R* Variant rs17782313 Associates With Increased Levels of DNAJC27, Ghrelin, and Visfatin and Correlates With Obesity and Hypertension in a Kuwaiti Cohort

**DOI:** 10.3389/fendo.2020.00437

**Published:** 2020-07-07

**Authors:** Maha M. Hammad, Mohamed Abu-Farha, Prashantha Hebbar, Preethi Cherian, Irina Al Khairi, Motasem Melhem, Fadi Alkayal, Osama Alsmadi, Thangavel Alphonse Thanaraj, Fahd Al-Mulla, Jehad Abubaker

**Affiliations:** ^1^Research Division, Department of Biochemistry and Molecular Biology, Dasman Diabetes Institute, Kuwait City, Kuwait; ^2^Research Division, Department of Genetics and Bioinformatics, Dasman Diabetes Institute, Kuwait City, Kuwait; ^3^Department of Human Genetics, McGill University, Montreal, QC, Canada; ^4^King Hussein Cancer Center, Amman, Jordan

**Keywords:** cAMP, DNAJC27, ghrelin, MC4R, obesity, visfatin

## Abstract

Melanocortin 4 receptor (MC4R), a notable component of the melanocortin system, regulates appetite, body weight, and energy homeostasis. Genome-wide association studies have identified several *MC4R* variants associated with adiposity; of these, rs17782313, which is associated with increased body mass index (BMI) and overeating behavior, is of particular interest. Another gene associated with increased adiposity in global genome-wide association studies is *DNAJC27*, a heat shock protein known to be elevated in obesity. The detailed mechanisms underlying the role of *MC4R* variants in the biological pathways underlying metabolic disorders are not well-understood. To address this, we assessed variations of rs17782313 in a cohort of 282 Arab individuals from Kuwait, who are deeply phenotyped for anthropometric and metabolic traits and various biomarkers, including DNAJC27. Association tests showed that the rs17782313_C allele was associated with BMI and DNAJC27 levels. Increased levels of DNAJC27 reduced the MC4R-mediated formation of cAMP in MC4R ACTOne stable cells. In conclusion, this study demonstrated an association between the rs17782313 variant near *MC4R* and increased BMI and DNAJC27 levels and established a link between increased DNAJC27 levels and lower cAMP levels. We propose that regulation of MC4R activity by DNAJC27 enhances appetite through its effect on cAMP, thereby regulating obesity.

## Introduction

Obesity has become a global epidemic and is increasing at an alarming rate worldwide, especially in the Arabian Peninsula region. Obesity can dramatically affect an individual's quality of life and is associated with increased risk of developing metabolic diseases, including diabetes, hypertension, and cardiovascular complications.

The leptin–melanocortin pathway is commonly dysregulated in obesity. The melanocortin system integrates neural, hormonal, and metabolic signals (1). Its action is mediated by melanocortins, a group of peptide hormones that include adrenocorticotropic hormone (ACTH), melanocyte-stimulating hormone (MSH), and Agouti-related peptide (AgRP) (2–4). These hormones can bind to and activate a group of melanocortin receptors (MCRs) that belong to the family of G protein-coupled receptors (GPCRs). To date, five melanocortin receptors (MC1R through MC5R) have been cloned and characterized and shown to have distinct distribution patterns and physiological functions (5). MC3R and MC4R, which are mainly expressed in the central nervous system, are often referred to as neural MCRs. Both play a key role in regulating energy homeostasis, as has been demonstrated using both agonists and antagonists for the receptors, especially for MC4R, confirming its physiological role. In addition, MC4R-knockout mice display obesity, hyperphagia, hyperinsulinemia, and hyperglycemia, providing further evidence that MC4R has a role in the regulation of energy homeostasis (2, 6, 7). The action of MC4R is mediated through two main populations of neurons that regulate feeding behavior: the anorexigenic pro-opiomelanocortin (POMC)/CART neurons and the orexigenic AgRP/neuropeptide Y (NPY) neurons. These neurons are expressed in the arcuate nucleus of the hypothalamus and function in opposition to each other. POMC is the prohormone for MSH and ACTH. POMC neurons project into the paraventricular hypothalamus (PVH) and release MSH, which in turn binds to and activates MC4R on the PVH neurons. This activation results in blocking the appetite and reducing food intake. Conversely, AgRP neurons project into the PVH releasing AgRP, which blocks MC4R. This results in the activation of appetite and an increase in food intake (1, 3, 8).

MC4R signaling is coupled to the three main heterotrimeric G proteins: Gs (stimuli), Gi (inhibition), and Gq (2, 4, 9, 10). When MC4R couples to Gs, this activates a cyclic adenosine monophosphate (cAMP)-dependent pathway (11), which results in the activation of adenylyl cyclase (AC) and the subsequent release of cAMP and the activation of protein kinase A (PKA). Conversely, when MC4R couples to Gi, this inhibits AC, whereas coupling to Gq results in the stimulation of phospholipase C (PLC), leading to the hydrolysis of phosphatidylinositol-4,5-bisphosphate (PIP2) into diacylglycerol (DAG) and inositol-1,4,5-triphosphate (IP3). IP3 results in the release of Ca^2+^ from intracellular stores while DAG activates PKC. MC4R signaling via G protein-independent pathways has also been demonstrated, including via the mitogen-activated protein kinase (MAPK) pathway to activate ERK1/2 and JNK phosphorylation, and through coupling to the inwardly rectifying potassium channel, Kir7.1 (12–14).

Multiple variants within and near the *MC4R* gene have been shown to be associated with metabolic diseases; indeed, these have been reported to be the most frequent genetic cause of obesity in humans, with more than 150 variants reported in patients with various ethnic origins (2, 10). Variants in *MC4R* have been reported in 3%−5% of early onset or severe adult obesity cases (15). MC4R has often been used as a target for obesity treatment because variants that result in decreased MC4R activity are associated with obesity (6, 16, 17). In addition, *MC4R* loss-of-function variants have been shown to affect an individual's ability to maintain weight reduction after exercise (18). A study on newborns showed that the variant rs17782313 is associated with changes in BMI over the first 2 weeks of life, and with body weight and BMI at the age of 2 weeks (19). Other genetic studies have suggested that some single nucleotide polymorphisms (SNPs) of *MC4R* influence the success of bariatric surgery (10, 20, 21). Furthermore, a recent study based on data from 0.5 million individuals in the UK Biobank demonstrated that gain-of-function *MC4R* variants were associated with protection against obesity (22). The *MC4R* rs17782313 variant is located 188 kb downstream of the gene and has been shown to have the strongest association with body mass index and obesity risk in a number of populations (23). Even though this variant is downstream of the *MC4R* gene by 188 kb, disruption of transcriptional control of the MC4R has been proposed as the likely functional mechanism for the variant (23). The GTeX database reports this variant as upregulating the expression of MC4R in tissues including testis, ovary, and brain (basal ganglia). The *MC4R* rs17782313 variant has also been shown to be associated with an increased risk of type 2 diabetes mellitus (T2D) in a meta-analysis that included more than 100,000 individuals; remarkably, this association was independent of BMI (24). There has been some inconsistency in the previous studies regarding this variant's role in diabetes and this could be attributed to discrepancy in study design, sample size and the population under investigation. It's also not very clear how it could be increasing the risk for developing diabetes. One possibility is that the *MC4R* gene is involved in regulating insulin resistance since animal studies revealed that MC4R knockout mice have 2-fold higher plasma insulin than their controls (25). However, further studies are still needed to understand this effect.

Additionally, data from the GWAS catalog showed that *MC4R* rs17782313-T was associated with height in Europeans (26) and East Asians (27) and *MC4R* rs17782313-C was associated with increased BMI (in Europeans) (23, 28), obesity (in Europeans, both children and adults) (29), physical activity measurement, and BMI (in African Americans and Europeans) (30).

The gene *DNAJC27/RBJ* has also been shown by GWAS to be associated with increased BMI (31). This gene is located near the adenylyl cyclase 3 (*ADCY3*) gene, which has been shown to interact with MC4R (32). DNAJC27/RBJ, a member of the heat shock protein (HSP) 40 family involved in the heat shock response pathway, plays an essential role in the pathology of insulin resistance and T2D. We recently demonstrated that DNAJC27 levels are elevated in PBMCs, plasma, and adipose tissue of individuals with obesity and T2D and that it is downregulated in obese participants after exercise (33, 34). We also reported that DNAJC27 was positively associated with obesity biomarkers such as leptin and resistin (34).

Little is known about the impact of *MC4R* variants on the biological pathways implicated in metabolic diseases or about the involvement of DNAJC27 protein, which could constitute an important target for understanding the functional role of MC4R in metabolic diseases. Given the important role of rs17782313 in regulating MC4R activity, the aim of this study was to evaluate the prevalence of this variant in a Kuwaiti cohort and to determine its role in regulating novel obesity biomarkers, especially DNAJC27.

## Materials and Methods

### Study Population

The study population comprised 282 participants resident in Kuwait. Their age, sex, comorbidities (such as diabetes and cardiovascular complications), baseline characteristics including height, weight, and waist circumference (WC) were recorded at enrolment. Participants with BMI > 30 kg/m^2^ were classified as obese. Diabetes and hypertension status were self-reported by participants. The study protocol was reviewed and approved by the Ethical Review Committee of Dasman Diabetes Institute and was conducted in accordance with the guidelines of the Declaration of Helsinki and the US Federal Policy for the Protection of Human Subjects. All participants signed an informed consent form before participating in the study.

### Sample Processing

The collection of blood samples was performed in accordance with established institutional guidelines. Participants were requested to fast for 8 h overnight prior to blood sample collection and samples were collected in the morning between 8 and 11 am. DNA was extracted using a Gentra Puregene kit (Qiagen, Valencia, CA, USA) and quantified using Quant-iT PicoGreen dsDNA Assay Kits (Life Technologies, Grand Island, NY, USA) and an Epoch Microplate Spectrophotometer (BioTek Instruments). Absorbance values at 260–280 nm were checked for adherence to an optical density range of 1.8–2.1.

### Anthropometric Measurements and Blood Biochemistry

The BMI of each participant was calculated as the ratio of their weight (kg) to height (m) squared. Fasting blood glucose (FBG) and lipid profiles, including triglyceride (TGL), low density lipoprotein (LDL), high density lipoprotein (HDL), and total cholesterol, were measured using a Siemens Dimension RXL integrated chemistry analyzer (Diamond Diagnostics, Holliston, MA, USA). HbA1c levels were determined using a Variant testing system (Bio-Rad, Hercules, CA, USA). C-peptide, leptin, adiponectin, ghrelin, and visfatin were measured using bead-based multiplexing technology on a Bio-Plex system (Bio-Rad, Hercules, CA, USA). We used the Human Diabetes 10-Plex kit (for C-peptide, leptin, ghrelin, and visfatin) and the 2-Plex Pro Human Diabetes kit (for adiponectin and adipsin) (Bio-Rad, Hercules, CA, USA). The average intra-assay coefficient of variation for all analytes was 4.25% with a range of 3.0–6.0%, and the average inter-assay coefficient of variation for all analytes was 3.75% with a range of 2.0–6.0%. HsCRP secreted levels were measured using an ELISA kit (Biovender, USA). The intra-assay coefficient of variation was 4.0–7.0%, and the inter-assay coefficient of variation was 5.5–6.3%.

### Genotyping

The TaqMan Genotyping Assay on an ABI 7500 Real-Time PCR System (Applied Biosystems, Foster City, CA, USA) was used to perform the candidate SNP genotyping. Each polymerase chain reaction sample comprised 10 ng of DNA, 5× FIREPol Master Mix (Solis BioDyne, Estonia), and 1 μl of 20× TaqMan SNP Genotyping Assay, with the thermal cycling conditions set at 60°C for 1 min and 95°C for 15 min, followed by 40 cycles of 95°C for 15 s and 60°C for 1 min. The genotypes determined by these techniques were confirmed for selected cases of homozygotes and heterozygotes by Sanger sequencing using a BigDye Terminator v3.1 Cycle Sequencing Kit on an 3730xl DNA Analyzer (Applied Biosystems, Foster City, CA, USA).

### Measurement of Plasma Levels of DNAJC27 Using ELISA

Plasma levels of DNAJC27 were measured using an ELISA kit (Wuhan EIAab Science Co., Wuhan, China). The plasma samples were thawed on ice and then centrifuged for 5 min at 10,000 × g at 4°C to remove any remaining cells or platelets. The samples were then diluted by a factor of four with sample diluent. ELISA was performed in accordance to kit instructions. Briefly, the samples and standards were loaded onto the assay plate and incubated for 2 h at 37°C, then washed and incubated for 1 h at 37°C, successively followed by the addition of the conjugated antibody and then streptavidin. Finally, the plate was incubated with TMB substrate for 30 min at 37°C, the reaction was stopped using acidic stop solution, and the absorbance was measured using a Synergy H4 plate reader at a wavelength of 450 nm. All the reagents used were provided in the kit. The intra-assay coefficient of variation was 3.0–5.0%, and the inter-assay coefficient of variation was 3.5–6.0%. According to the manufacturer's manual, recovery was determined by spiking various levels of DNAJC27 into serum and plasma and they reported 92% recovery in serum and 94% recovery in plasma.

### Cell Culture

MC4R ACTOne stable cells (e.Enzyme, Gaithersburg, Maryland, USA) which are a HEK-293-CNG cell line expressing a recombinant human MC4R and a modified cyclic nucleotide gated channel that opens in response to elevated intracellular cAMP levels were used in all *in vitro* assays. They were grown in Dulbecco's modified Eagle medium supplemented with 10% fetal bovine serum, 250 μg/ml G418, and 1 μg/ml puromycin. The cells were plated on 10-cm dishes 24 h prior to transfection. All experiments were performed on 75–80% confluent plates. Transient transfections were performed using Lipofectamine 2000, following the manufacturer's instructions, with 3 μg of each construct. An empty pCMV vector was used as a control. pCMV and DNAJC27 were purchased from Origene (Herford, Germany). The expression vector for DNAJC27 is pCMV. At 18 h post-transfection, 70,000 cells were re-seeded into 96-well plates coated with poly-D-lysine and left to attach overnight. A membrane potential assay was performed the following day.

### cAMP Formation

cAMP formation was measured using a membrane potential assay kit (e.Enzyme, Gaithersburg, Maryland, USA), following the manufacturer's instructions. The cells were starved in Dulbecco's phosphate-buffered saline for 1 h at 37°C and then incubated with 1X dye-loading solution for 2 h at room temperature in the dark. The cells were then stimulated with increasing concentrations of NDP-α-MSH (Sigma, Taufkirchen, Germany) or setmelanotide (MedChemExpress, Sollentuna, Sweden) for 10 min at 37°C. Setmelanotide was used because of its specificity to MC4R. The fluorescence was measured with a Synergy H4 plate reader with excitation at 530/20 nm and emission at 590/20 nm. The percentage of cAMP formation was calculated after correcting for the non-stimulated cells and normalizing to the maximum response to the highest agonist concentration (3 μM).

### Quality Assessment of SNP

The PLINK genome association analysis toolset version 1.9 was used to assess the quality and statistical association of the rs17782313 SNP. Quality assessments including minor allele frequency (MAF) and consistency with the Hardy–Weinberg equilibrium were performed for the *MC4R* variant. Any quantitative trait values <Q1–1.5 × the interquartile range (IQR) or >Q3+1.5 × IQR were considered to be outliers and excluded from the statistical analysis.

### Statistical Analysis

Data are expressed as mean ± standard deviation (SD) and comparisons were performed using Student's *t*-test for quantitative variables and Fisher's exact test for categorical variables to determine significance. *P* ≤ 0.05 were significant. Allele-based associations between the rs17782313 variant and the three binary traits of disease status (obesity, diabetes, and hypertension) were evaluated using a logistic regression model adjusted for age and sex. In this model, the logarithm of the odds of each disease was used as response variable, with additive combinations of the explanatory variables such as alleles and the covariates of age and sex in the models as its predictors to estimate probability of minor allele being risk for disease. The risk of disease status due to the effect allele was assessed by the odds ratio and standard error of the odds ratio. Further, allele-based associations between the rs17782313 variant and each of the quantitative traits were evaluated using a linear regression model adjusted for age and sex. In this model, quantitative trait was used as response variable, with additive combinations of the explanatory variables such as alleles and any covariates (age, sex) in the model as its predictors. The change in the mean of phenotype measurement was assessed by regression coefficient (Beta), where a positive regression coefficient means that the minor allele increases risk effect. Associations were considered statistically significant when the *P* ≤ 0.05.

## Results

### Characteristics of the Study Participants and Genotyping Data

The rs17782313 variant near *MC4R* has previously been reported to be associated with obesity in various populations, but not yet in a Kuwaiti population. We therefore investigated the presence of this polymorphism in a population of 282 people living in Kuwait. Table 1 presents an overview of the study population and their clinical and anthropometric parameters. The minor allele frequency (MAF) for the *MC4R* loci in our sample for rs17782313 was 28%. The average rate of successful genotyping across this SNP was >99%, and all the markers were in Hardy–Weinberg equilibrium. Among the 282 samples that were genotyped, 22 (7.8%) were homozygote for C, 146 (51.77%) were homozygote for T, and 114 (40.43%) were heterozygote (TC).

**Table 1:** Overview of study population as per genotype distribution at the *MC4R* variant rs17782313.

**Traits**	**Measurements Mean (±SD)**	**TT Mean (±SD)**	**TC+CC Mean (±SD)**	***P*-value^$^**
Sex (Male: Female) %	45.2: 54.8	42.4: 57.6	48.1: 51.9	0.339
Obesity status (Yes: No) %	48.6: 51.4	43.75: 56.25	53.7: 46.3	0.118
Diabetes status (Yes: No) %	43.2: 56.8	39.6: 60.4	47: 53	0.227
Hypertension status (Yes: No) %	30.7: 69.3	26.4: 73.6	35.3: 64.7	0.119
Age (years)	46.25 (±12.38)	46.58 (±12.31)	45.9 (±12.51)	0.648
BMI (kg/m^2^)	29.9 (±5.18)	29.39 (±5.12)	30.52 (±5.12)	0.069
Cholesterol (mmol/l)	5.02 (±1.09)	4.983 (±1.13)	5.068 (±1.06)	0.523
TGL (mmol/l)	1.23 (±0.59)	1.161 (±0.58)	1.3 (±0.61)	0.059
LDL(mmol/l)	3.13 (±0.96)	3.089 (±0.94)	3.176 (±0.99)	0.462
HDL (mmol/l)	1.2 (±0.32)	1.210 (±0.33)	1.192 (±0.31)	0.659
Glucose (mmol/l)	5.78 (±1.24)	5.705 (±1.25)	5.859 (±1.23)	0.336
HbA1C (%)	6.31 (±1.30)	6.215 (±1.21)	6.423 (±1.37)	0.202
C-peptide (pg/ml)	2.67 (±1.73)	2.413 (±1.67)	2.931 (±1.76)	0.057
HsCRP (μg/ml)	5.328 (±0.48)	5.290 (±0.509)	5.367 (±0.457)	0.217
Leptin (pg/ml)	7,193.4 (±4,138.21)	7,238 (±4,516)	7,145 (±3,712)	0.885
Adiponectin (ng/ml)	4,294.2 (±2,497.67)	4,412 (±2,548)	4,166 (±2,448)	0.475
**Ghrelin (pg/ml)**	546.9 (±215.72)	508 (±207.1)	586.8 (±218.4)	**0.0197**
**Visfatin (pg/ml)**	3,681.9 (±1,154.08)	3,440 (±1,131)	3,946 (±1,128)	**0.0051**
**DNAJC27 (ng/ml)**	4.63 (±3.23)	3.655 (±2.47)	5.598 (±3.60)	**0.0003**

$ *Significant P-values are shown in bold font. Comparisons were performed using Student's t-test for quantitative variables and Fisher's exact test for categorical variables to determine significance. P ≤ 0.05 were significant*.

**Figure 1:**
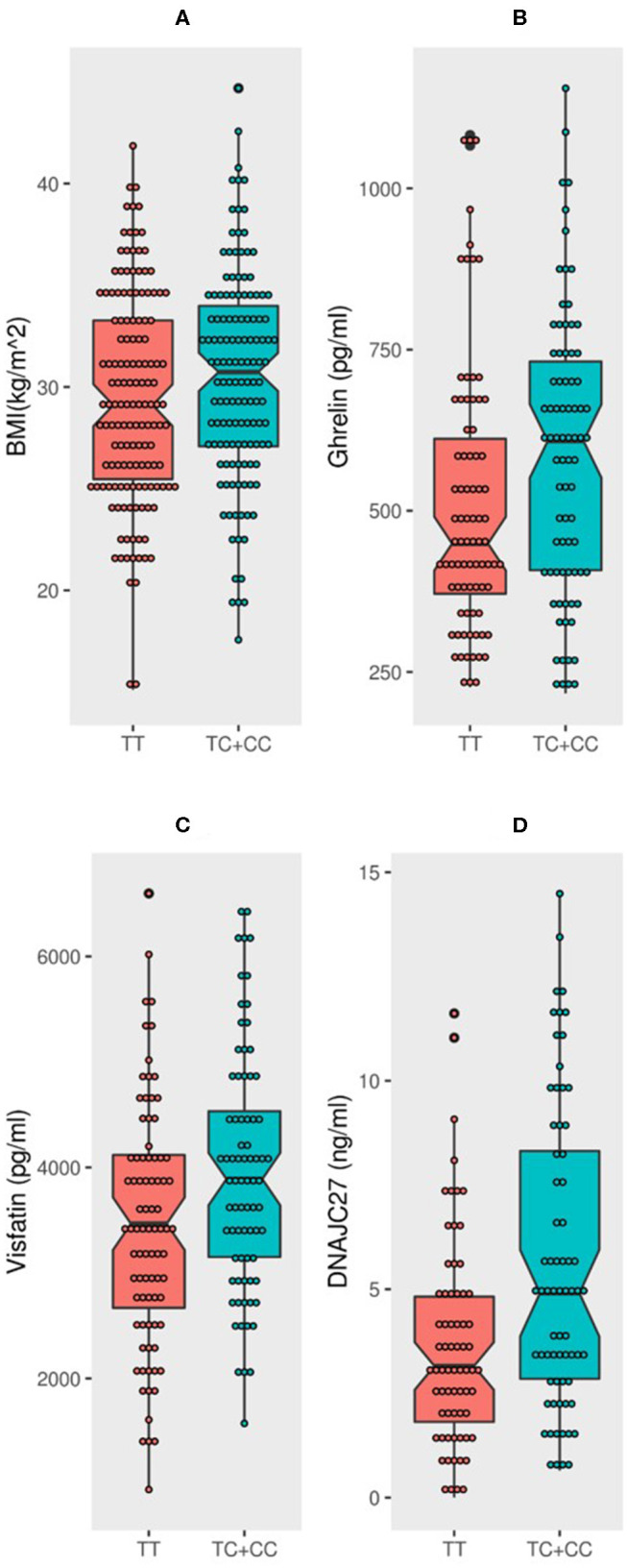
*MC4R* rs17782313 carriers have significantly higher BMI, DNAJC27, ghrelin, and visfatin than non-carriers: Boxplot analysis of **(A)** BMI, **(B)** Ghrelin, **(C)** Visfatin, and **(D)** DNAJC27 plasma levels in participants based on their genotype.

The population was classified into two groups based on their genotyping profile: SNP non-carriers (TT) and SNP carriers (TC+CC). The mean BMI was marginally higher in the participants that harbored the C allele than in the non-carriers (30.52 ± 5.12 kg/m^2^ vs. 29.39 ± 5.19 kg/m^2^; *P* = 0.0691) (Figure 1A). Table 2 summarizes the results of allele-based association tests that assessed the risk for obesity in individuals carrying the effect allele (C allele). This showed that the C allele was significantly associated with the obesity status of the participants with an odds ratio of 1.5 and a *P*-value of 0.05. Furthermore, the C allele was seen associated with the hypertension status of the participants with an odds ratio of 1.6 and a *P*-value of 0.04.

**Table 2:** Allele-based association tests to assess risk for disease status in individuals carrying the effect allele (C allele).

**Disease status**	**Odds ratio (OR)**	**Confidence interval (CI) 95%**	**Standard error of the odds ratio**	***P*-value**
**Obese status**	1.46	0.994–2.15	0.1967	**0.0534**
Diabetes status	1.115	0.737–1.68	0.2107	0.6061
**Hypertension status**	1.605	1.01–2.53	0.2326	**0.0419**

*Logistic regression model was adjusted for age and sex. Significant P-values are shown in bold font*.

The participants with the carrier genotypes had significantly higher levels of some obesity markers. Ghrelin levels were significantly higher in those with the TC or CC genotypes compared to those with the TT genotype (586.8 ± 218 pg/ml and 508 ± 207 pg/ml, respectively; *P*-value = 0.0197) (Figure 1B). Similarly, the SNP carriers (TC+CC) had higher levels of visfatin than the TT carriers (3,946 ± 1,128 pg/ml vs. 3,440 ± 1,131 pg/ml; *P*-value = 0.0051) (Figure 1C). Interestingly, we observed a highly significant increase in plasma DNAJC27 levels in the rs17782313 carriers compared to the non-carriers (5.598 ± 3.602 ng/ml vs. 3.655 ± 2.473 ng/ml; *P*-value = 0.0003) (Figure 1D). The levels of leptin and adiponectin were comparable between the rs17782313 carriers and non-carriers (Table 1) and no association was observed between the SNP and the two hormones (Table 3).

**Table 3:** Results of association tests between the rs17782313 and the quantitative phenotype traits. Linear regression model was adjusted for age and sex.

**Traits (*n*)^@^**	**Effect Size**	***P*-value**
**BMI (kg/m^2^)** **(277)**	0.9673	**0.0458**
Cholesterol (mmol/l) (272)	0.0373	0.7211
TGL (mmol/l) (259)	0.08245	0.1298
LDL(mmol/l) (268)	0.03882	0.6777
HDL (mmol/l) (259)	−0.00254	0.9305
Glucose (mmol/l) (240)	0.07222	0.5143
HbA1C (%) (253)	0.08778	0.4566
C-peptide (pg/ml) (161)	0.4063	0.0654
HsCRP (ng/μl) (240)	14680	0.6515
Leptin (pg/ml) (167)	398.9	0.3322
Adiponectin (ng/ml) (210)	−213.6	0.4095
**Ghrelin (pg/ml)** **(162)**	49.35	**0.0504**
**Visfatin (pg/ml)** **(161)**	359.3	**0.0095**
**DNAJC27 (ng/ml)** **(138)**	1.406	**0.0029**

@ *The samples sizes differ for each trait because of the quality control procedures for checking outliers in trait measurements and missing information. Significant P-values are shown in bold font*.

### Association Between rs17782313 Near *MC4R* and Obesity-Related Markers

The association tests for the variant and traits or markers showed that four traits were significantly associated with the *MC4R* rs17782313 variant (Table 3): BMI (*P*-value = 0.046), DNAJC27 (*P*-value = 0.003), ghrelin (*P*-value = 0.05), and visfatin (*P*-value = 0.009).

### Effect of DNAJC27 Expression on MC4R-Mediated cAMP Formation

**Figure 2:**
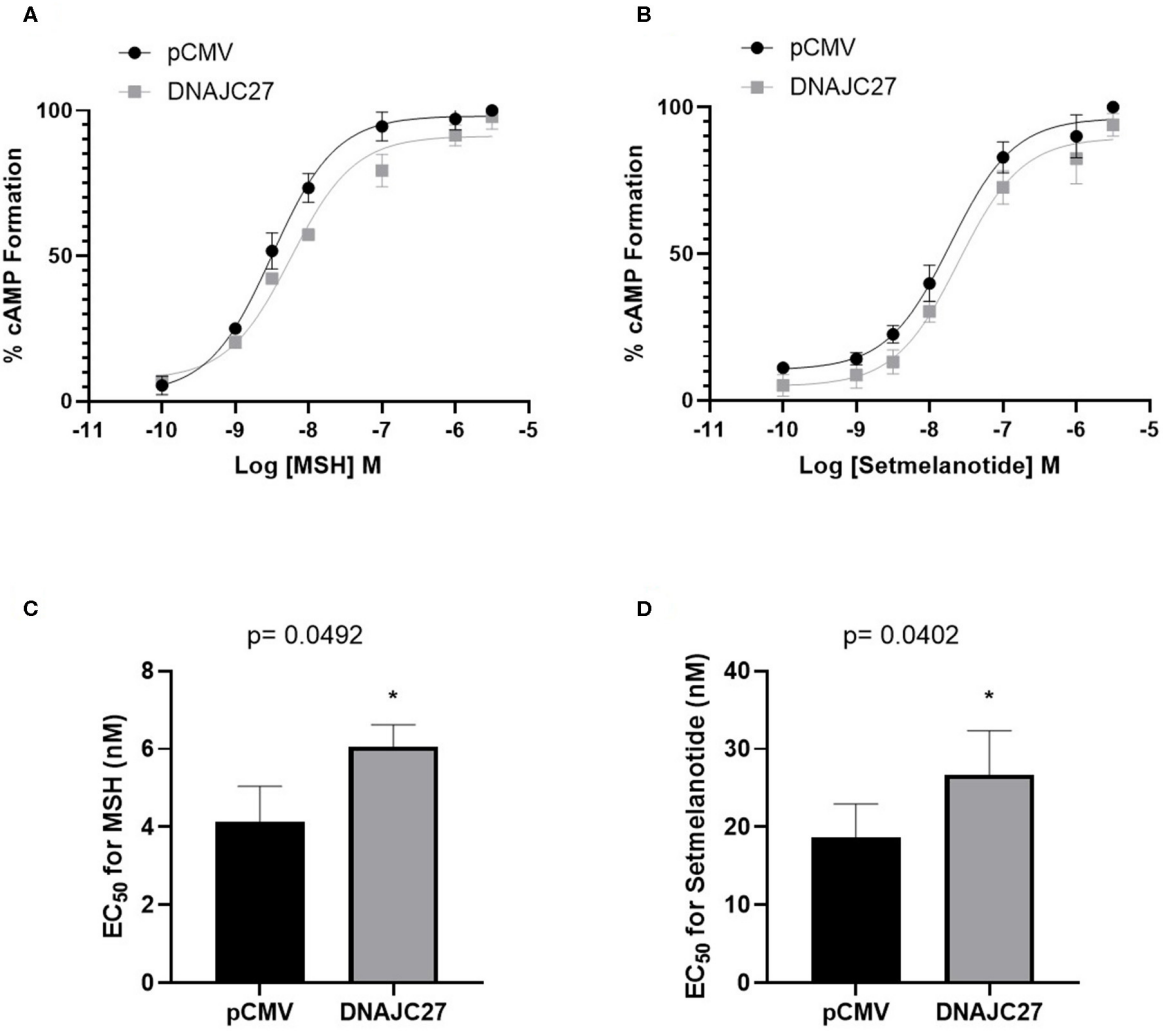
Overexpression of DNAJC27 can reduce MC4R-mediated cAMP formation: Dose response for MC4R-stimulated cAMP formation assessed using membrane potential assay. **(A)** MSH and **(B)** Setmelanotide-mediated cAMP formation in either pCMV or DNAJC27- transfected HEK-MC4R cells. EC_50_ values from stimulation with **(C)** MSH or **(D)** Setmelanotide. Data are representative of three independent experiments. *P ≤ 0.05 vs. empty vector control.

To elucidate the association between DNAJC27 and MC4R and the underlying mechanism, we investigated the effect of the overexpression of DNAJC27 on MC4R signaling. We performed membrane potential assay to measure the formation of cAMP in the stable HEK-MC4R cell line transiently transfected with either pCMV (as an empty vector control) or DNAJC27 and treated for 10 min with increasing concentrations of NDP-α-MSH. When DNAJC27 was overexpressed, there was a significant reduction in the efficacy of NDP-α-MSH-stimulated cAMP signaling shown by a rightwards shift in the dose–response curve (Figure 2A) and higher EC_50_ value of 6.06 nM in the DNAJC27-transfected cells compared to 4.08 nM in the control pCMV-transfected cells (Figure 2C). Similar results were observed when setmelanotide (another selective MC4R agonist, also known as BIM-493 and RM-493), was used (Figures 2B,D); in this case, the EC_50_ value for the DNAJC27-transfected cells was 26.72 nM compared to 18.62 nM for the control pCMV-transfected cells.

## Discussion

The results of this study showed that the rs17782313 variant near *MC4R* was associated with hypertension, obesity, and high plasma levels of ghrelin and visfatin in a cohort of Arabic participants. The variant was also associated with elevated plasma DNAJC27/RBJ levels. In an independent experiment using cell lines, we have tested the impact of increased DNAJC27 on MC4R. In this cell line model, we showed that the overexpression of DNAJC27 resulted in decreased cAMP production by the *in vitro* activation of wild-type MC4R.

Several studies have reported the association of *MC4R* variants with obesity in various ethnic groups (23, 28–30). Recent association studies have begun to investigate *MC4R* polymorphisms in Arabs. A study in 2019 showed that *MC4R* rs17782313 was associated with obese polycystic ovary syndrome in the Western region of Saudi Arabia (35), and another study from Saudi Arabia showed rs17782313 to be associated with moderate obesity (36). The mechanism for such associations is usually discussed in terms of inducing appetite (37); it has also been proposed that appetite-determining genes might have a greater effect in Arab individuals (38). The present study showed a modest increase in BMI in individuals with carrier genotypes of the rs17782313 variant near *MC4R*, although the allele-based association tests showed a significant risk for obesity, with an odds ratio of 1.5. The *MC4R* rs17782313 variant was also significantly associated with BMI. These observations are notable considering that most of the study participants were classified as obese (the mean BMI of the participants was 29.9 ± 5.18 kg/m^2^). Our findings support the results of the previous studies on *MC4R* variants and reveal a novel association between DNAJC27 levels and the *MC4R* variant.

In the 1000 Genomes Project populations, the minor allele frequency of the rs17782313_T>C variant was 0.24. Data from the PAGE study (39) showed a variation in frequency across different populations, with the highest value of 0.36 observed in South Asians and the lowest value of 0.109 observed in Central Americans. The allele frequency of the variant among the participants in the present study was 0.2801, which is comparable to that previously reported for Africans (0.278) and African Americans (0.2795). In our cohort, TGL levels were higher in the participants with the carrier genotypes (TC+CC) compared to those with the TT genotype at close to statistical significance with borderline *P*-value of 0.0586. It would be interesting to investigate further the association between MC4R and lipid profile in a larger population, especially because previous studies have demonstrated that the *MC4R* rs17782313 variant was associated with visceral and subcutaneous fat distribution in a Chinese population (40) and a Polish population (41). Furthermore, a mechanistic study that explored the effect of lipid stress on MC4R trafficking in hypothalamic neuronal cells showed that treatment with palmitate inhibited the clathrin-mediated endocytosis of MC4R and impaired its desensitization (42). The present study also showed strong associations between the *MC4R* rs17782313 variant and the obesity-related proteins ghrelin and visfatin. The participants who carried the MC4R genotypes (TC+CC) had significantly higher levels of ghrelin and visfatin compared to those carrying the TT genotype. The increase in ghrelin levels was expected since this variant is associated with obesity and probably affects the functionality of MC4R which is known to regulate appetite. In fact, the GTeX database reports the rs17782313 variant as upregulating the expression of MC4R in tissues including testis, ovary, and brain. Since both ghrelin and visfatin are also obesity-related hormones, it is not surprising to see them at higher circulating levels in individuals with the carrier genotypes. Several studies suggest that MC4R regulates glucose homeostasis and insulin resistance (2, 43), therefore, visfatin might be involved in this regulation but further studies are still required to understand the mechanism governing those MC4R functions.

Taken together, our results support a potential role of the *MC4R* rs17782313 variant in hypertension and obesity. The role of ghrelin in the hypothalamic control of appetite is well-established since ghrelin decreases the activity of POMC neurons and increases the activity of AgRP neurons (3, 44). However, and to the best of our knowledge, no previous studies have investigated the effect of MC4R function on the secretion of ghrelin or visfatin. There are also no reports of an association between *MC4R* rs17782313 and ghrelin or visfatin levels. Only few studies have evaluated the impact of carrier genotypes on the levels of the markers examined in our study. For example, Brodowski et al. (45) reported significantly higher triglyceride levels in *MC4R* polymorphism carriers (C/X genotype) compared to those with the TT genotype in a population of non-morbid premenopausal women with obesity. Arrizabalaga et al. (46) reported no difference in serum ghrelin levels between those who carried the *MC4R* polymorphism (TC+CC) compared to those with the TT genotype. These discrepancies with our results may be due to ethnic differences as well as study design and population. For example, both previous studies limited the participants to females and while Brodowski et al. investigated postmenopausal women, Arrizabalaga et al. looked at premenopausal women. However, further studies with larger cohorts are needed for a more conclusive outcome.

In our previous studies of DNAJC27 in the context of obesity and T2D (33, 34), we demonstrated a positive association between DNAJC27 and the common obesity biomarkers leptin and resistin, and also that DNAJC27 levels were elevated in PBMCs, plasma, and adipose tissue from individuals with obesity and T2D. In the present study, the participants carrying the *MC4R* rs17782313_C allele had significantly higher plasma levels of DNAJC27 than the other participants. Because of the novelty of this finding, as well as the location of the *DNAJC27* gene in close proximity to *ADCY3*, a known regulator of the MC4R response, we investigated the effect of DNAJC27 on MC4R activity, measured by cAMP formation. The overexpression of DNAJC27 in HEK-MC4R cells reduced the efficacy of the MC4R-mediated formation of cAMP by two MC4R agonists, NDP-α-MSH and setmelanotide. Previous studies have reported that the activity of MC4R is a key modulator of food intake; stimulating MC4R induced satiety whereas blocking MC4R increased the appetite (1, 3, 8). Furthermore, loss-of-function genetic variants of *MC4R* are associated with obesity whereas gain-of-function genetic variants are protective against obesity (22). Our findings suggest that higher levels of DNAJC27 can reduce wild-type MC4R signaling *in vitro*, however, we still cannot make a direct link between the circulating levels of DNAJC27 and MC4R signaling in the hypothalamus and further studies are now required to understand how DNAJC27 could affect the appetite and the eventual development of obesity.

DNAJC27/RBJ has previously been shown to have a general effect in regulating the MAPK pathway through the accumulation of active MEK1/2 in the nucleus, which results in the activation of ERK1/2 (47). MC4R can also signal through the MAPK pathway, leading to ERK1/2 phosphorylation. This highlights the importance of studying the effect of DNAJC27 on the multiple signaling pathways activated by MC4R. These pathways eventually determine the physiological effects of the receptor and the functions it mediates in different tissues and disease contexts. For example, coupling to the G protein Gs and cAMP formation controls energy homeostasis, whereas ERK1/2 activation mediates insulin signaling and food intake.

Our findings showed that cAMP formation decreased when DNAJC27 was overexpressed, but the underlying mechanism for this remains unclear. Given that DNAJC27 belongs to the heat shock protein (HSP) family, and that HSPs are known to function as molecular chaperones in regulating many of the essential aspects in the life cycle of GPCRs (48, 49), we can speculate that DNAJC27 may bind to MC4R. This is supported by the overlapping tissue distribution of MC4R and DNAJC27, especially in the brain. One possibility is that DNAJC27 regulates the folding of MC4R. This in turn affects the subcellular trafficking of the receptor; misfolded receptors are polyubiquitinated and targeted for degradation by the proteasome while correctly folded receptors are transported to the Golgi apparatus for post-translational modifications and eventually fused into the cell membrane. DNAJC27 might also be enhancing the internalization of the receptor. Generally, the various functions of molecular chaperones can have a critical effect on the surface expression of GPCRs and hence mediate their downstream signaling cascades. Interestingly, a recent study in mice explored the effect of glucose-regulated protein 78 (GRP78) on the regulation of MC4R trafficking and signaling, showing an interaction between MC4R and GRP78 in hypothalamic protein extracts (50). The study also showed that knocking down endogenous GRP78 in the paraventricular nucleus (PVN) of the hypothalamus in a diet-induced obesity mouse model resulted in a significant increase in body weight. Another study investigating the role of Hsc70 and HSP90 in regulating MC4R expression reported that overexpression of these HSPs released the retained mutant forms of the receptor to the cell surface (51). Therefore, further studies using animal models are needed to determine the exact mechanism of crosstalk between MC4R and DNAJC27 and whether DNAJC27 functions as a molecular chaperone for the receptor.

It is important to point out here that although heat shock proteins are mainly thought of as intracellular proteins, many studies are showing members of the family to be detected in human serum and plasma. Moreover, HSP40 proteins are known to interact with HSP70 which has been well-documented to be expressed in plasma. The studies are also associating the differential expression of these circulating proteins with various diseases. For example, serum levels of DNAJB9 were shown to be elevated in fibrillary glomerulonephritis patients (52). HSP70 together with HSP60 were shown to be associated with cardiovascular diseases and inflammation. Specifically, while circulating HSP70 levels are elevated in patients after acute myocardial infarction (53) and were shown to be predictive of acute coronary syndrome (54), HSP60 levels are involved in hypertension and atherosclerosis (55). Several studies also investigated the effect of exercise on modulating the circulating levels of HSPs (HSP27, HSP70, and HSP72) and generally found them to be increased (56, 57).

One of the limitations of this study is the cross-sectional design as well as lack of data on the levels of DNAJC27 in the hypothalamus of individuals who carry the C-allele. However, this study demonstrated the strong association between the C allele at the rs17782313 polymorphic locus of the *MC4R* gene and obesity, hypertension, and elevated plasma levels of DNAJC27. Taken together, the findings of the study suggest that overexpressed DNAJC27 reduces cAMP formation by wild-type MC4R, and this provides a potential mechanism of action for the effect of elevated circulating DNAJC27 on appetite and eventually the development of obesity.

## Data Availability Statement

The datasets generated for this study are available on request to the corresponding author.

## Ethics Statement

The studies involving human participants were reviewed and approved by Ethical Review Committee of Dasman Diabetes Institute. The patients/participants provided their written informed consent to participate in this study.

## Author Contributions

JA, TT, and FA-M designed, supervised, helped in writing, and reviewed the manuscript. MH, MA-F, and PH wrote the manuscript and supervised the experiments. IA, MM, FA, and PC performed the experiments. OA analyzed the data and critically reviewed the manuscript. All authors contributed to the article and approved the submitted version.

## Conflict of Interest

The authors declare that the research was conducted in the absence of any commercial or financial relationships that could be construed as a potential conflict of interest.
